# Peer Review of “Real-World Performance of COVID-19 Antigen Tests: Predictive Modeling and Laboratory-Based Validation”

**DOI:** 10.2196/83476

**Published:** 2025-10-06

**Authors:** Helena de Puig

**Affiliations:** 1 Wyss Institute for Biologically Inspired Engineering, Harvard University, Boston, MA, United States

**Keywords:** COVID-19, real-world data, limit of detection lateral flow test, probability of positive agreement, logistic regression, COVID-19 antigen test clinical performance

*This is a peer-review report for “Real-World Performance of COVID-19 Antigen Tests: Predictive Modeling and Laboratory-Based Validation.”*

## Round 1 Review

This is a good paper [1] that models the performance of antigen tests.

### Minor Comments

1. It would be good to include a schematic/analysis/methods and an image of how the lateral flow assays look and how test band intensities are obtained. Was that done with ImageJ?
2. An interesting aspect of the paper is the comparison between trained eye versus user of lateral flow assays (Figure 5). I think that adding a paragraph about the conclusions from that figure might be good in the Discussion section.
